# Association of LA volumes and mid-wall myocardial fibrosis in the Dilated cardiomyopathy patients

**DOI:** 10.1186/1532-429X-13-S1-P289

**Published:** 2011-02-02

**Authors:** Fariba Fanaie, Ankur Gulati, Andrew Jabbour, Banya Winston, Sanjay Prasad, Raad Mohiaddin

**Affiliations:** 1 Royal Brompton Hospital, London, UK

## Introduction

Left atrial (LA) size as assessed by echocardiography has been shown to have prognostic value in patients with dilated cardiomyopathy (DCM). More recently cardiac magnetic resonance (CMR) imaging studies have reported that the presence of left ventricular mid-wall late gadolinium enhancement (LGE), a marker of myocardial fibrosis, also predicts adverse outcome in DCM.

The biplane area-length method has been validated as an accurate method for assessment of LA volumes, which echocardiography may underestimate by up to 47% compared with CMR.

## Purpose

We sought to establish whether LA volume as assessed by CMR was associated with the presence of mid-wall LGE in DCM patients.

## Methods

DCM patients were studied between Jan 2006-Aug 2008. Patients with coronary artery disease were excluded. All patients underwent a comprehensive CMR study which included acquisition of LGE images in 2 separate phase-encoding directions to exclude artefact. LA area and length were traced at end-ventricular systole and diastole in both horizontal and vertical long axis images. Maximum (LA max) and minimum (LA min) LA volumes were then calculated using the biplane area-length method for ellipsoid bodies and indexed to BSA. A separate cardiologist who was blinded to the results of the LA area determined the presence of mid-wall LGE.

## Results

One hundred and ninety two patients (152 male; age 50.7 years ± 14.7 years (mean±SD); LV EF 40% ± 15%, were recruited to the study. The overall mean indexed LA max was 54.5 (95% CI: 51.7-57.5) and mean indexed LA min 29.8 (95%CI: 23.7-27.1).

Patients with concurrent mid-wall LGE had significantly larger indexed LA max volumes than those without LGE (56.2 ml (25.6 ml - 60.9 ml) vs. 49.7 ml (45.5 ml -54.2 ml) (mean (95%CI), p = 0.03)). Patients with concurrent LGE also had significantly larger indexed LA min volumes than those without (31.9 ml (28.6 ml - 35.7 ml) vs. 24.3 ml (20.7 ml - 28.5 ml) respectively; p < 0.01). The differences remained statistically significant when patients with atrial fibrillation were excluded from analysis.

## Conclusion

LA volumes were significantly greater in DCM patients with left ventricular mid-wall LGE compared to those with no LGE. The presence of mid-wall LGE may therefore be associated with increased left atrial pressures in DCM patients. Further studies are therefore required to evaluate whether LA size has incremental prognostic value to midwall LGE in DCM.

